# Supplementary material for: Reversion of a RND transporter pseudogene uncovers latent stress resistance in Brucella ovis
Source: bioRxiv. 2025 May 10:2025.05.10.653276. Preprint. [Version 1] doi: 10.1101/2025.05.10.653276 (PMC12248096; doi:10.1101/2025.05.10.653276)
Supplement: Supplement 1 [file media-1.pdf]

Supplemental Tables and Figures

Table S1. Compound hits identified from high-throughput screening of the Prestwick Chemical library. These compounds passed the initial screening criteria as selectively inhibitory to *B. ovis* during THP-1 macrophage infection with limited axenic activity.

Sample	Compound name	Therapeutic class	Major pathway or target
MSU-51	Trichlorfon	Pesticide	Acetylcholinesterase inhibitor
MSU-339	Guanfacine hydrochloride	Hypertension	Adrenergic receptor agonist
MSU-270	Fendiline hydrochloride	Antiarrhythmic	Calcium
MSU-368	Bepridil hydrochloride	Angina	Calcium
MSU-383	Nicardipine hydrochloride	Hypertension	Calcium
MSU-1264	Lomerizine hydrochloride	Migraine	Calcium
MSU-1188	Cilnidipine	Hypertension	Calcium
MSU-1180	Bifonazole	Antimicrobial	Cell membrane (fungi)
MSU-1011	Flucloxacillin sodium	Antimicrobial	Cell wall
MSU-470	Ceforanide	Antimicrobial	Cell wall
MSU-700	Cefmetazole sodium salt	Antimicrobial	Cell wall
MSU-1212	Ezetimibe	Cholesterol absorption	Cholesterol
MSU-370	Benzbromarone	Gout	Cytochrome P450
MSU-605	Carbadox	Antimicrobial	DNA synthesis
MSU-350	Clozapine	Antipsychotic	Dopamine
MSU-360	Droperidol	Nausea	Dopamine
MSU-374	Methylergometrine maleate	Uterine atony	Dopamine
MSU-980	Piribedil hydrochloride	Depression	Dopamine
MSU-1163	Aripiprazole	Antipsychotic	Dopamine, serotonin
MSU-626	Racecadotril	Diarrhea	Enkephalinase
MSU-976	Tracazolate hydrochloride	Sedative	GABA
MSU-457	Meclozine dihydrochloride	Nausea	Histamine
MSU-589	Azelastine HCl	Allergic conjunctivitis	Histamine
MSU-888	Promethazine hydrochloride	Allergy	Histamine
MSU-1260	Ritonavir	HIV	HIV protease
MSU-973	Pirlindole mesylate	Depression	Monoamine oxidase
MSU-173	Tranylcypromine hydrochloride	Depression	Monoamine oxidase
MSU-144	Loperamide hydrochloride	Diarrhea	Mu-opioid receptors

MSU-581	Reboxetine mesylate	Depression	Noradrenaline
MSU-1211	Ipriflavone	Osteoporosis	Osteoclast
MSU-376	Clofazimine	Leprosy	Peroxisome
MSU-587	Cilostazol	Vasodilator	Phosphodiesterase
MSU-977	Zardaverine	Cancer	Phosphodiesterase
MSU-142	Dipyridamole	Anticoagulant	Phosphodiesterase
MSU-1031	Halofantrine hydrochloride	Malaria	Porphyrin
MSU-533	Phenacetin	Analgesia	Prostaglandin
MSU-660	Avermectin B1a	Antimicrobial	Protein synthesis
MSU-476	Primaquine diphosphate	Malaria	Reactive oxygen species
MSU-1105	Verteporfin	Macular degeneration	Reactive oxygen species
MSU-590	Etretinate	Psoriasis	Retinoic acid
MSU-531	Pirenperone	Anxiety	Serotonin
MSU-979	Ozagrel hydrochloride	Thrombosis	Thromboxane
MSU-853	Liothyronine	Hypothyroidism	Thyroid
MSU-494	Propylthiouracil	Hyperthyroidism	Thyroid peroxidase
MSU-381	Lidoflazine	Experimental	Unknown
MSU-421	Suloctidil	Experimental	Unknown
MSU-550	Parthenolide	Dermatitis	Unknown
MSU-1013	Deptropine citrate	Experimental	Unknown
MSU-1054	Levopropoxyphene napsylate	Cough	Unknown

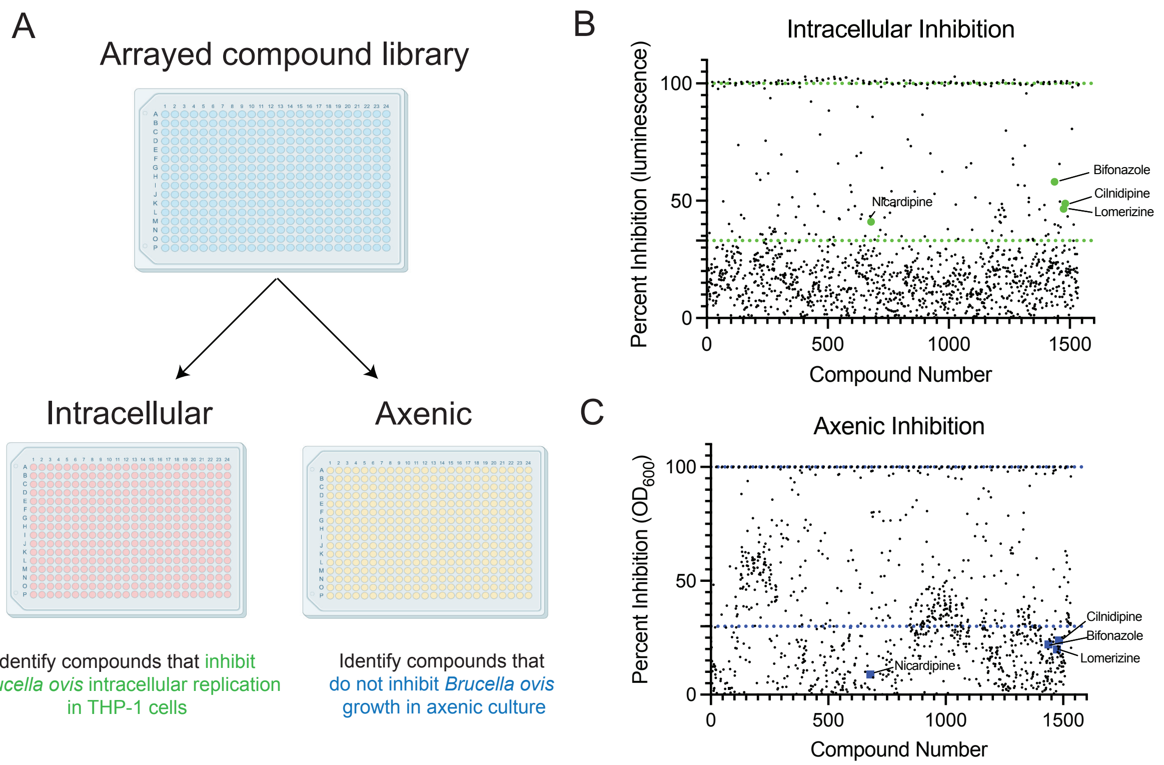

Figure S1. Complementary small-molecule screens identify compounds that selectively inhibit *B. ovis* intracellular growth in THP-1 macrophage-like cells. (A) Diagram of the drug screening pipeline for the identification of small molecules that selectively inhibit *B. ovis* intracellular growth with minimal axenic activity. (B) Intracellular inhibition of all tested small molecules, shown as percentage of luminescence emitted by *B. ovis* cells harboring the *lux* operon. Highlighted in green are drug candidates that inhibited *B. ovis* intracellular growth in THP-1 macrophages. The screening of the Prestwick Chemical library had a Z' factor of 0.417 for inhibition of intracellular growth. Dotted line represents hit determination of 35% intracellular inhibition. (C) Effect of small molecules on *B. ovis* growth inhibition in axenic culture. The screening of the Prestwick Chemical library had a Z' factor of 0.473 for axenic growth inhibition. Dotted line represents 35% axenic inhibition. The compounds highlighted in green in B and blue in C are hits that inhibited *B. ovis* intracellular growth but had minimal axenic activity based on our screening criteria.

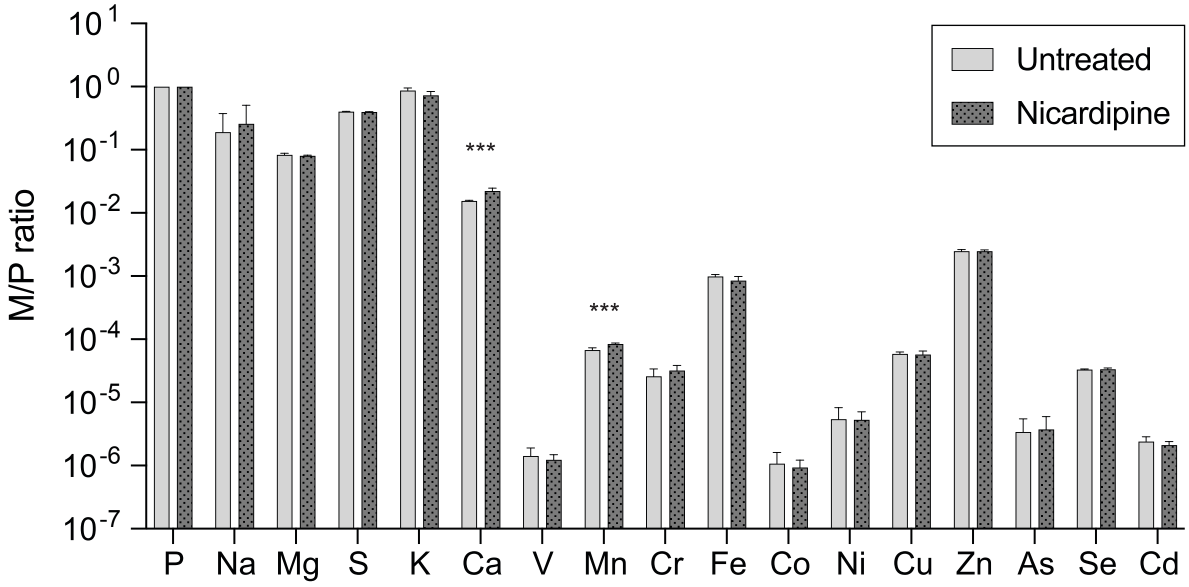

Figure S2. Full elemental profile of THP-1 untreated or treated with 25 μM nicardipine for 48 hours. Element content was determined by triple quadrupole inductively coupled plasma mass spectrometry (ICP-QQQ). Levels of each element were normalized to total phosphorus levels (M/P). Bars represent the mean ± standard deviation of 7 biological replicates measured over 2 independent experiments. The M/P ratios for each metal were compared using multiple unpaired t-tests and the Bonferroni-Dunn method to adjust for multiple comparisons (***, adjusted $P < 0.001$).

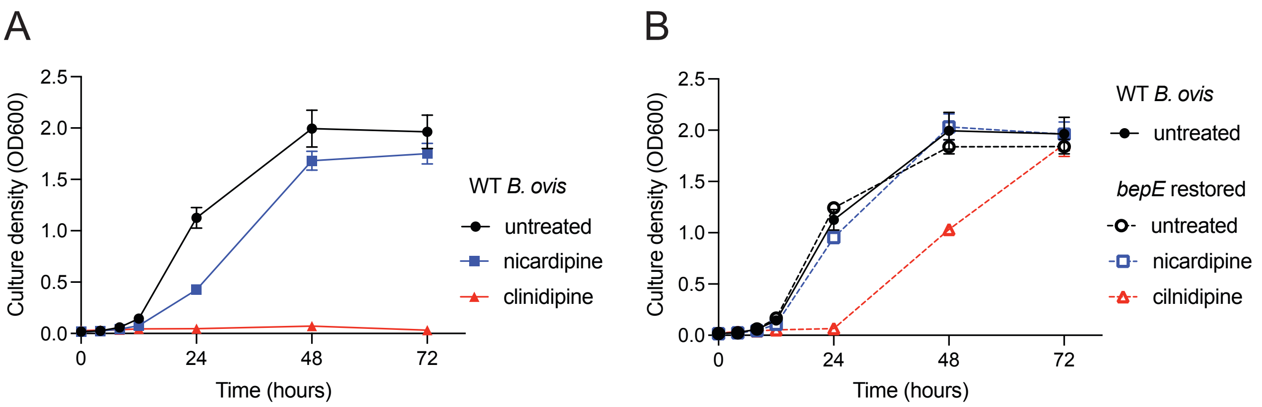

Figure S3. Restoration of *B. ovis bepE* confers resistance to dihydropyridine calcium channel blockers during growth in liquid culture. (A) Growth of wild-type (WT) *B. ovis* cultures, untreated (black) 25 μM nicardipine (blue) or 25 μM cilnidipine (red), was monitored by optical density at 600 nm. (B) Growth of the *bepE* restored strain in the same treatments as in (A). The WT untreated culture is presented in both panels for reference.

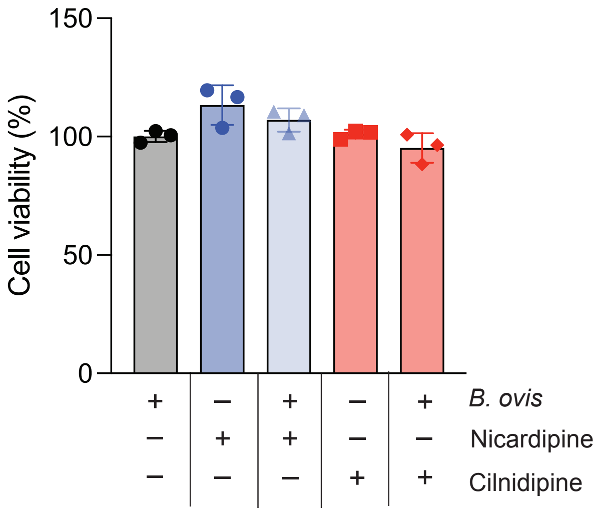

Figure S4. *B. ovis* infection and treatment with calcium channel blockers does not affect THP-1 host cell viability. Viability of THP-1 cells, assessed at 48 h post infection or following treatment with 25 μM nicardipine or 25 μM cilnidipine with the XTT-cell proliferation assay. Viability was normalized to wells containing untreated THP-1 cells infected with *B. ovis* and to wells only containing cell culture medium (blank). Values are means ± SD from three independent trials.

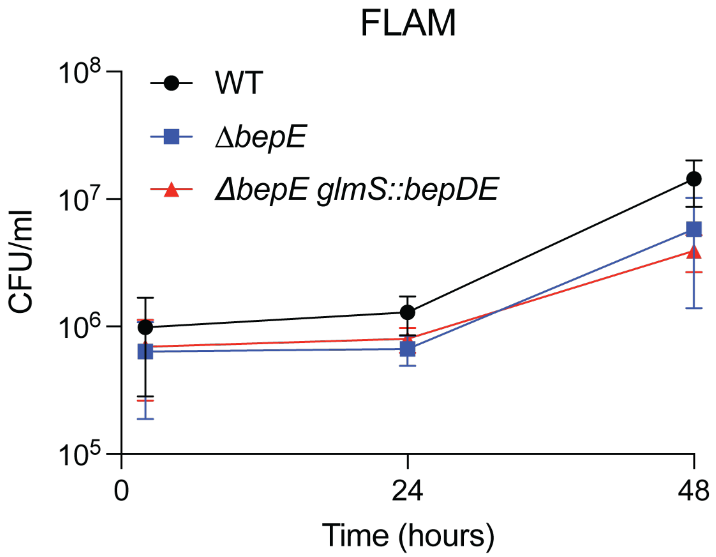

Figure S5. *bepE* does not contribute to *B. abortus* survival in fetal liver alveolar macrophages (FLAM). *B. abortus* (WT, Δ*bepE*, and the complementation strain Δ*bepE glmS::bepDE*) recovered from FLAM cells after infection. Values are means ± SD CFU recovered from three independent trials.

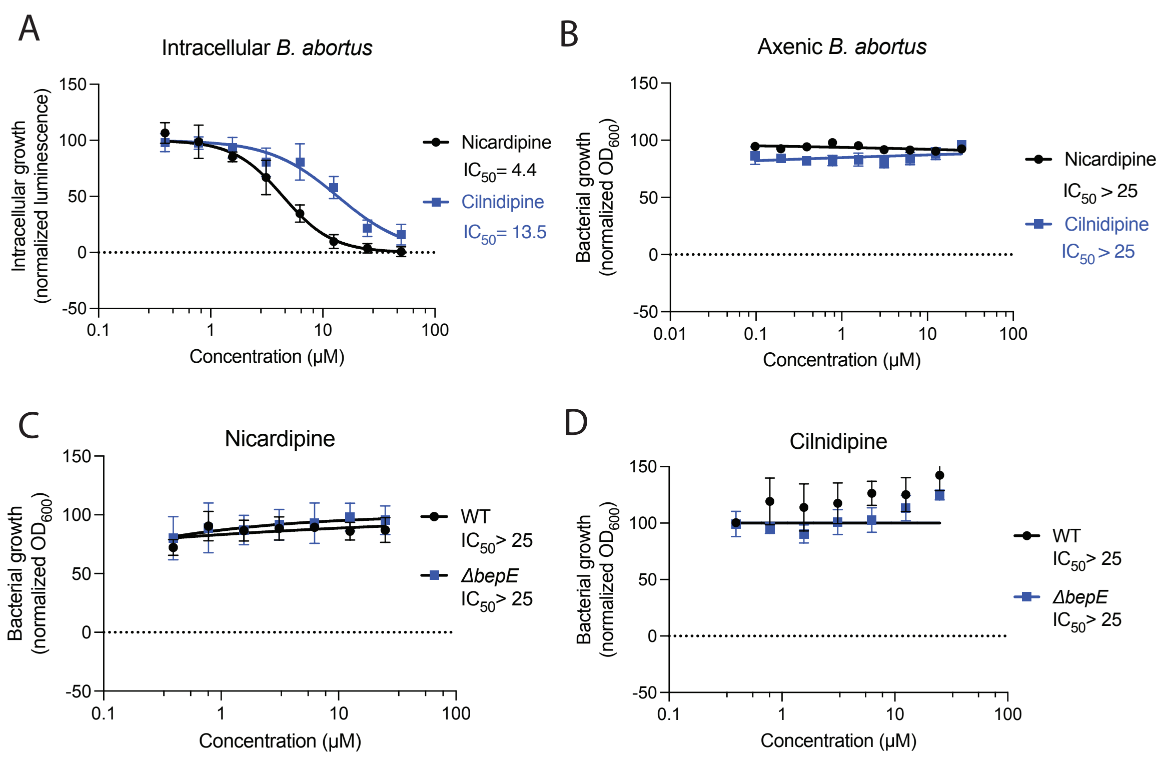

Figure S6. Nicardipine and cilnidipine are potent inhibitors of intracellular, but not axenic growth of *B. abortus*. (A) Intracellular inhibitory activities of nicardipine or cilnidipine during infection of THP-1 macrophages by *lux* expressing *B. abortus*. Luminescence was measured after 48 h and normalized to untreated infected controls. (B) Axenic inhibitory activity of nicardipine and cilnidipine during *B. abortus* WT growth in liquid medium. Optical density at 600 nm was measured at 48 h and normalized to untreated cultures. (C, D) Disruption of *bepE* does not affect axenic sensitivity of *B. abortus* to nicardipine or cilnidipine. Growth was measured and analyzed as in panel B.
